# Cereal Aphid Colony Turnover and Persistence in Winter Wheat

**DOI:** 10.1371/journal.pone.0106822

**Published:** 2014-09-30

**Authors:** Linton Winder, Colin J. Alexander, Chris Woolley, Joe N. Perry, John M. Holland

**Affiliations:** 1 Department of Natural Sciences, Unitec Institute of Technology, Auckland, New Zealand; 2 Biomathematics and Statistics Scotland, The James Hutton Institute, Perth and Kinross, Invergowrie, United Kingdom; 3 School of Animal, Plant and Environmental Sciences, University of the Witwatersrand, Johannesburg, Gauteng, Republic of South Africa; 4 Plant and Invertebrate Ecology, Rothamsted Research, Harpenden, Herts, United Kingdom; 5 Farmland Ecology Unit, Game and Wildlife Conservation Trust, Fordingbridge, Hants, United Kingdom; University College Dublin, Ireland

## Abstract

An understanding of spatial and temporal processes in agricultural ecosystems provides a basis for rational decision-making with regards to the management and husbandry of crops, supporting the implementation of integrated farming strategies. In this study we investigated the spatial and temporal distribution of aphid pests (*Sitobion avenae* and *Metopolophium dirhodum*) within winter wheat fields. Using an intensive sampling programme we investigated distributions at both the small (single shoot) and large (field) scales. Within two fields, a grid with 82 locations was established (area 120 m by 168 m). At each location, 25 shoots were individually marked and aphid counts by observation conducted on 21 and 22 occasions as the crop matured, resulting in 43,050 and 45,100 counts being conducted in the two fields respectively. We quantified field scale spatial distributions, demonstrating that spatial pattern generally emerged, with temporal stability being both species- and field- dependent. We then measured turnover of colonies at the small (individual shoot) and large (field) scales by comparing consecutive pairs of sampling occasions. Four turnover categories were defined: Empty (no aphids recorded on either occasion); Colonised (aphids recorded on the second occasion but not the first); Extinction (aphids recorded on the first occasion but not the second); Stable (aphids recorded on both occasions). At the field scale, population stability soon established, but, at the small scale there was a consistently high proportion of unoccupied shoots with considerable colonisation and extinction and low stability. The redistribution of aphids within the crop at the local scale is a vulnerability which could be used to disrupt population development – by mediating exposure to ground-active natural enemies and by incurring a metabolic cost caused by the physiological demands to re-establish on a nearby host plant.

## Introduction

An understanding of spatial [1] and temporal [2] processes are crucial in order to understand the complexities of ecosystems. In agricultural systems such understanding is needed because it supports rational decision-making with regards to the management and husbandry of crops [3]; such information can assist in the effective implementation of integrated farming strategies and in particular the targeted application of fertilisers, fungicides and pesticides. In this intensive study, we investigated the spatial and temporal characteristics of cereal aphids, a common and sometimes serious crop pest. Cereal aphids affect grain quality [4], cause yield loss through direct damage [5], and act as virus vectors [6].

Within a cereal field the aphid population is characteristically spatially patchy, both at the small [7] and the field scale [8]–[10]. Knowledge regarding such spatial distributions and how they develop through time is important because such information may be used to optimise insecticide usage through selective spraying [11]. Additionally, spatial pattern mediates interactions between aphids and their natural enemies [12], [13] and therefore impacts on the delivery of the ecosystem service of biological control [14]. An understanding of within-field aphid distributions could therefore support the optimisation of landscape characteristics that enhance distributions of beneficial insects [15]–[17].

Although the spatial distribution of cereal aphids at the field-scale has been described in a number of studies, few have addressed processes at the local (i.e. individual shoot) scale. An exception is the detailed study conducted by Fievet et al. [7], which showed that aphid colonies experience high rates of local extinction and colonisation (i.e. turnover), presumably due to dispersal and predation [18]. Local-scale dispersal has been shown to influence the transmission rate of viruses [19] and increase the vulnerability of individual aphids to predation by ground-active predators or web-building spiders that would otherwise be unable to prey upon them [20]–[23]. Cereal aphids are known to be highly mobile; typically 20–35% disperse through falling from the plant host day^−1^ [24]–[27].

This intensive field study investigated the spatial and temporal distribution of cereal aphids within winter wheat. The objective of the study was to characterise, through time, both spatial pattern and turnover (i.e. extinction or colonisation) of aphid colonies at the local and field scales.

## Materials and Methods

### The field sites

**Figure 1:**
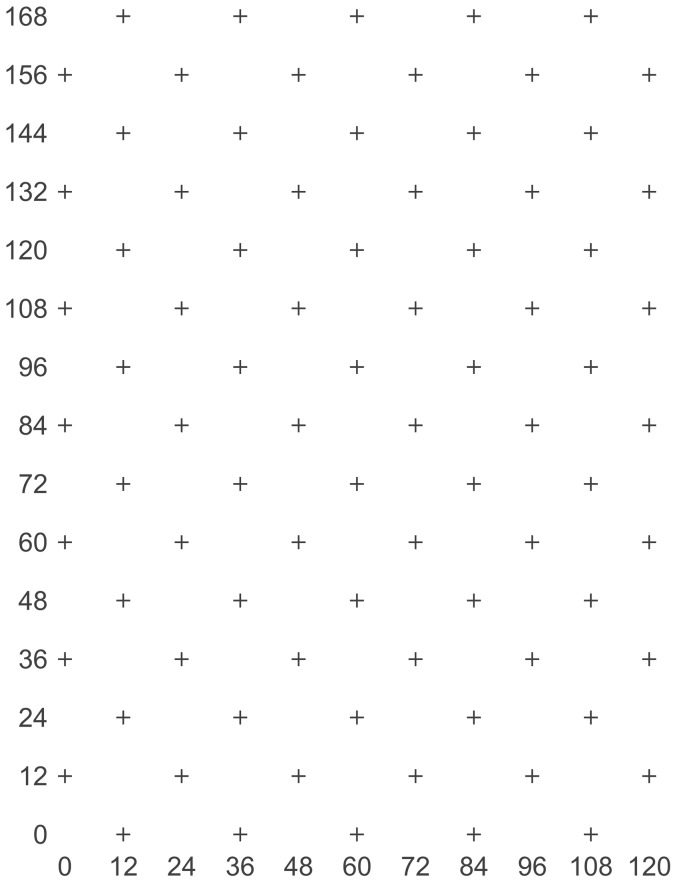
Offset sampling grid used in both fields sampled during the study.

Field work was conducted on Seale-Hayne farm privately owned by the University of Plymouth (Newton Abbot, TQ12 6NQ) and did not involve endangered or protected species. No other locations were utilised during the study. Seale-Hayne is now owned by the Dame Hannah Rogers Trust Registered Charity Number 306948. The Dame Hannah Rogers Trust should be approached for future permission to conduct field work at Seale-Hayne. The study was conducted within two conventionally managed winter wheat fields located on Seale-Hayne farm, and was part of a series of studies conducted simultaneously [27]. The two fields (A and B) were 8.1 ha and 5.4 ha respectively, and sown in October 2001 with the variety ‘Claire’. Insecticides were not applied to either field during the study. A sampling grid with 82 locations was established within each field in an offset pattern within an area of 120 m by 168 m (Figure 1). During the season crop growth stage was recorded using the Zadoks scale [28]. Each location was identified by a flexible cane extending beyond crop height with a waterproof card bearing location number. A narrow path was cut from tractor wheelings to each sampling location in order to prevent lodging of the crop by trampling during repeated sampling.

### Intensive assessment of aphid populations

We intensively monitored the distribution of cereal aphids: at each of the 82 sampling locations within each field, 25 shoots were individually marked (within a 1 m^2^ area adjacent to the flag) using small waterproof cards. Aphid counts by direct observation were conducted on each shoot (i.e. the stalk, leaves and once emerged, the ear), recording species and number of individuals. Counts were taken in dry weather conditions on 21 occasions in Field A and 22 occasions in Field B from mid-May to late July. The sampling interval was usually three or four days, but this was varied if poor weather prevented counts from being taken. This represented a substantial sampling effort; 43,050 and 45,100 individual aphid counts were conducted in Field A and Field B respectively during the study (count data summarised in Table S1 and Table S2). The recorded aphid counts were then used to assess the within-field populations, assess population turnover and characterise observed spatial distributions. Firstly, summary statistics (mean number of aphids shoot^−1^ and the proportion of shoots infested) were generated.

Secondly, spatially explicit analysis of these data was done using Spatial Analysis by Distance Indices (SADIE) utilising ‘red-blue’ plot and ‘local association’ methodology [10], [29], [30]. Red-blue analysis (a spatially referenced visualisation of spatial pattern) provided a means of measuring the presence of field-scale spatial pattern by identifying neighbourhoods of consistently high counts (patches) or consistently low counts (gaps) within each field. Each location was designated an x,y coordinate and a corresponding count *c* (derived by summing the 25 individual counts) for each sampling occasion was calculated. Using SADIE methodology, each location was ascribed an index of clustering in relation to the mean field count *m*: either a positive *v*_i_ index for patch units with *c*_i_>*m*, or a negative *v*_j_ index for gap units with *c*_j_<*m*. These cluster indices were then used to calculate an overall cluster index 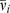 and 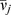 (with associated significance values) that indicated whether the field was characterised by the presence of measurable patches, gaps or both. We used SadieShell version 2.0 (available for download at http://home.cogeco.ca/~sadiespatial/index.html) for all analyses and used the SADIE non-parametric option (to account for variances exceeding the mean).

Using SADIE procedures, pairwise comparisons of sampling dates were done to determine whether aphid patches or gaps persisted through time. This was done by testing for local spatial association using N_AShell (version 1, also downloadable). Local spatial association measured the cluster index similarity between two sampling dates and was measured using the index χ_k_. This statistic is based on the similarity between the clustering indices drawn from the two data sets. χ_k_ was positive if local association was evident due to similarity of the two cluster indices (i.e. *v*_k_/*v*_k_ or -*v*_k_/-*v*_k_; patch-patch or gap-gap coincidence) and negative if local dissociation was present (i.e. *v*_k_/-*v*_k_ or -*v*_k_/*v*_k_; patch-gap or gap-patch coincidence) at a given location respectively. An overall spatial association statistic X was calculated from the mean of these local values, equivalent to a simple correlation coefficient. This statistic was positive when overall local association was evident and negative for overall local dissociation [10]. The method provided a formal test of significance; for association P was <0.025 (indicating that aphid colonies persisted locally between consecutive sample dates), whilst P was>0.975 for dissociation. A non-significant result indicated that no local association between the two spatial distributions could be detected. It should be noted that measurable local association does not necessarily imply that field-scale spatial pattern was evident; a temporally stable but spatially random distribution would exhibit spatial association between sample dates. To demonstrate temporally stable field-scale spatial pattern, both the red-blue spatial parameters and local association should in fact be measurable. These data were generated and represented in a correlation matrix surface created in Surfer 10 (Golden Software). Local association analysis required multiple pairwise testing and so consideration of the issue of multiple comparisons was addressed. Rather than use a Bonferroni approach [31] that alters the P value, we conducted a preliminary analysis and tabulated the possible combinations (210 for Field A and 232 for Field B) noting how many were significant. We then compared our results to the expected Type I error at the 5% level (i.e. 10.5 for Field A and 11.6 for Field B) to determine whether the set of association values were showing a real study effect. Our preliminary analysis indicated that the number of significant associations was considerably higher than that expected by chance. Results were (for count-based analysis): for Field A 56 and 35 significant for *S. avenae* and *M. dirhodum* respectively with an expectation of 10.5 due to Type I error, and for Field B 29 and 28 significant for *S. avenae* and *M. dirhodum* respectively with an expectation of 11.6 due to Type I error. We therefore accepted the validity of the analyses but nonetheless interpreted them conservatively (by focussing on consistency of pattern rather than the significance of individual pairwise comparisons).

Thirdly, an analysis was done to measure turnover of colonies on individual shoots (local turnover) and turnover of sampling locations (field-scale turnover) following the approach described by Fievert et al. [7]. Turnover was investigated by comparing each pair of consecutive sample dates and four states were defined:

1. Empty. No aphids recorded on either sample date.
2. Colonised. Aphids recorded on the second sampling occasion but not the first.
3. Extinction. Aphids recorded on the first sampling occasion but not the second.
4. Stable. Aphids recorded on both sampling occasions.

For the sampling location analysis (field-scale turnover), aphids were considered present if at least one of the 25 shoots at a location had individuals present. These records were used to calculate the proportion of sampling locations within each of the four categories for each pair of consecutive sample dates. For the individual shoot (local turnover) analysis, the proportion of the 25 shoots within each of the four categories was calculated for each sampling location, and then the average proportion of shoots within each category was then determined for the field as a whole for each pair of consecutive sample dates.

## Results

Two cereal aphid species were recorded within the fields, the English grain aphid *Sitobion avenae* (Fabricius, 1775) and the rose grain aphid *Metopolophium dirhodum* (Walker, 1849). The populations of *S. avenae* developed and peaked following ear emergence and flowering, whilst *M. dirhodum* populations developed earlier and peaked at flowering.

### Field A

**Figure 2:**
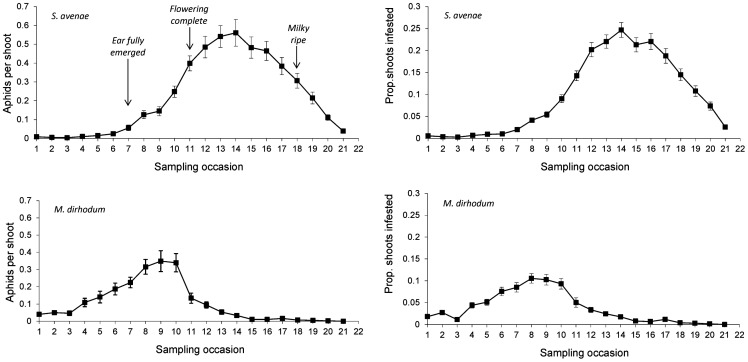
Population growth of *S. avenae* and *M. dirhodum* observed in Field A, summarised as aphids per shoot (mean ± 1 s.e.) and as proportion of shoots infested. Sampling occasions were: **1**, 19 May; **2**, 22 May; **3**, 26 May; **4**, 31 May; **5**, 2 June; **6**, 5 June; **7**, 9 June; **8**, 13 June; **9**, 16 June; **10**, 19 June; **11**, 23 June; **12**, 27 June; **13**, 28 June; **14**, 30 June; **15**, 3 July; **16**, 5 July; **17**, 7 July; **18**, 10 July; **19**, 14 July; **20**, 17 July; **21**, 21 July.

**Figure 3:**
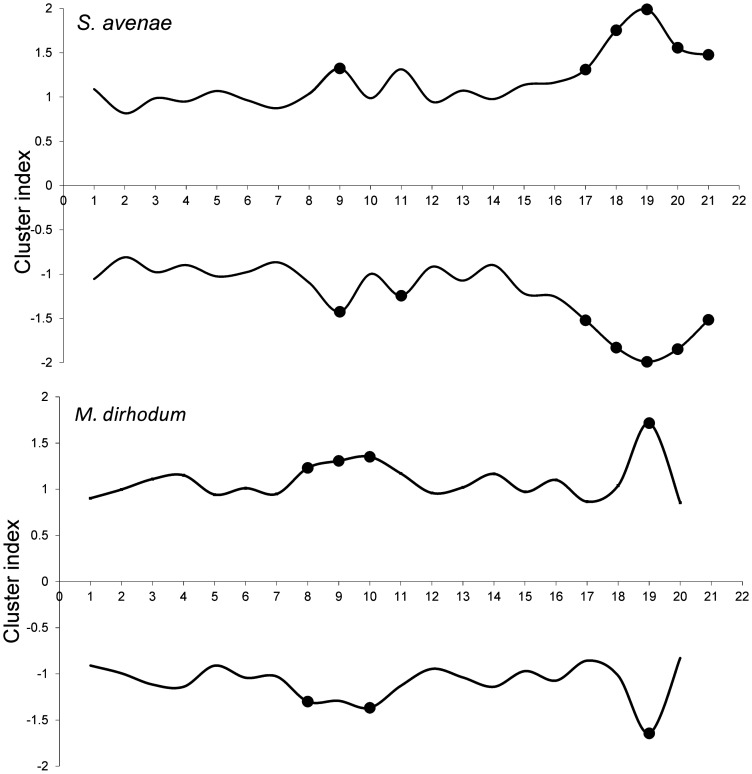
SADIE analysis representing patch (positive) and gap (negative) cluster analyses for *S. avenae* and *M. dirhodum* recorded in Field A. Filled circles represent sampling occasions when cluster indices were significant at 5% and represented measurable spatial pattern.

**Figure 4:**
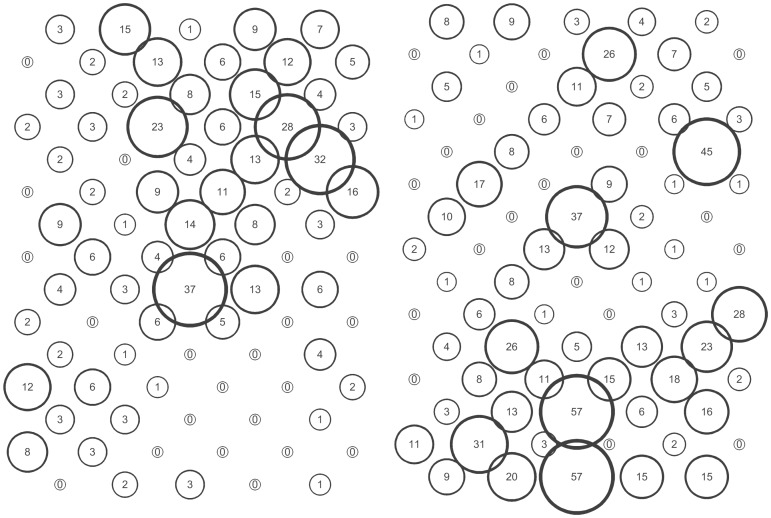
Indicative distributions in Field A when field-scale spatial pattern was most strongly expressed for *S. avenae* (sampling occasion 19, left) and *M. dirhodum* (sampling occasion 10, right).

**Figure 5:**
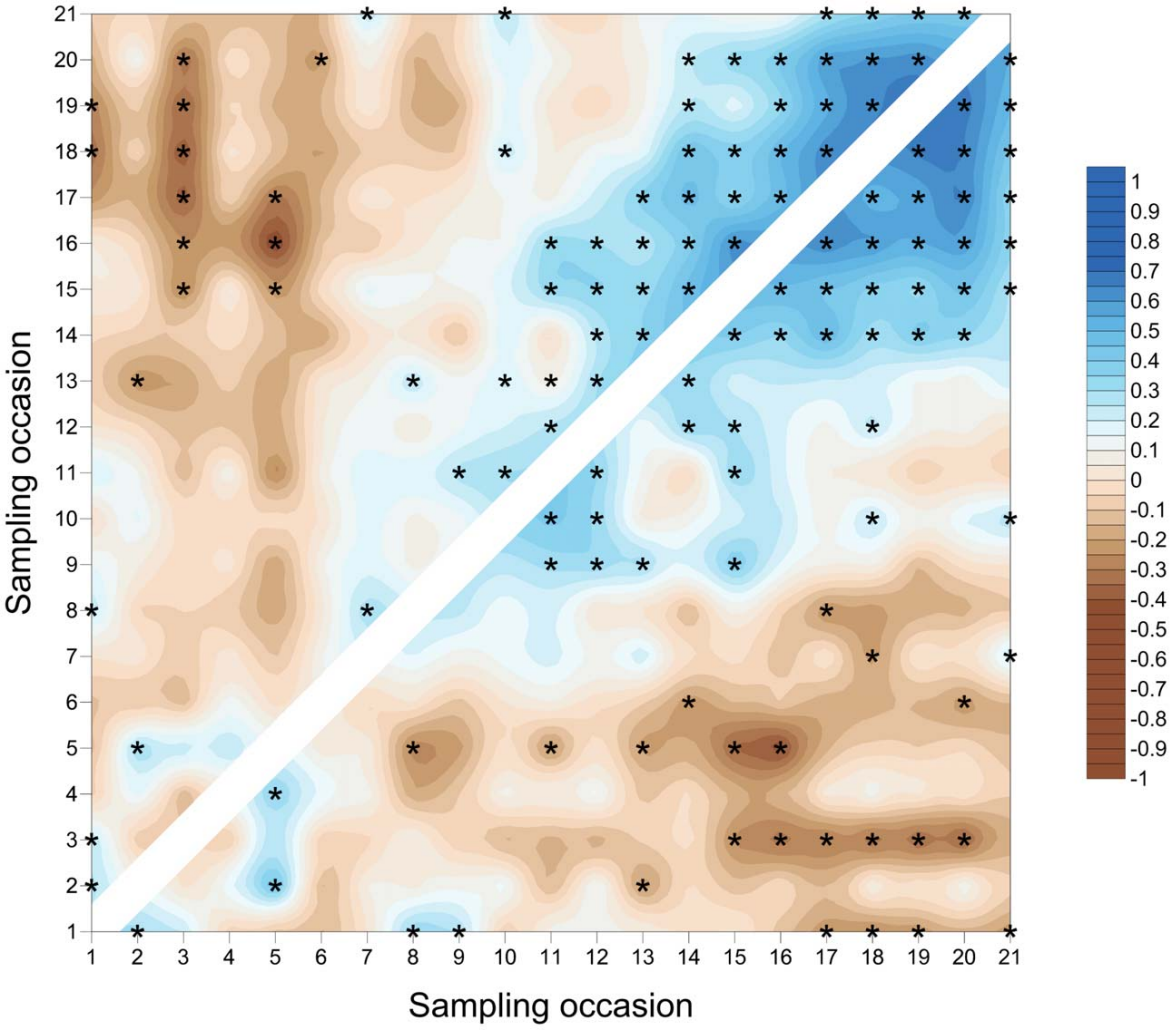
SADIE local association plot for *S. avenae* in Field A. Plot represents strength of local (pairwise) association between sampling occasions; blue colouring represents association and brown dissociation respectively. Significant local association at 5% is represented by asterisks. Comparisons assessed using counts (above diagonal) and proportion of shoots infested (below diagonal).

**Figure 6:**
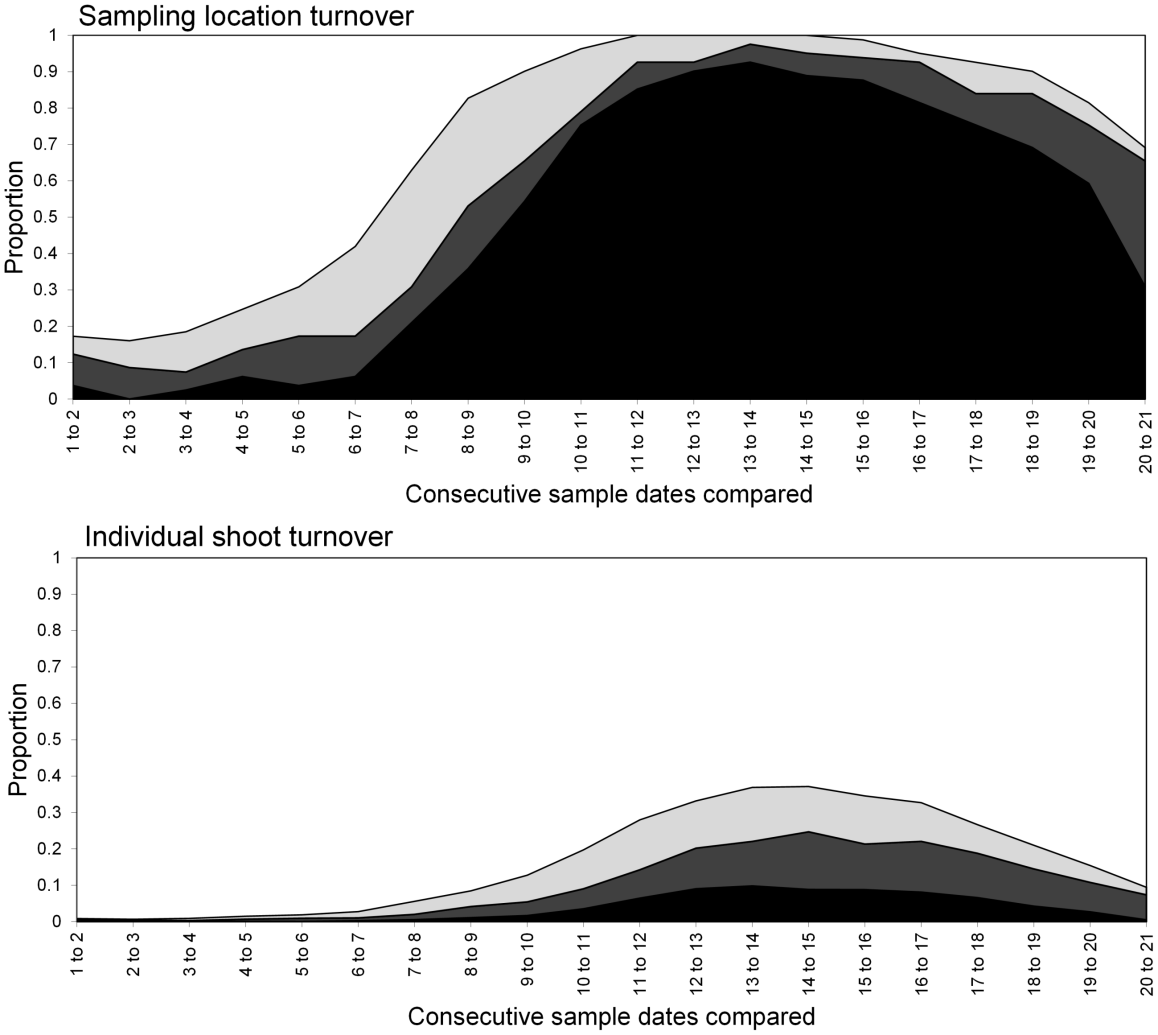
Turnover of *S. avenae* in Field A at sampling locations (upper) and individual shoots (lower) respectively. States represented as: stable (black), colonised (dark grey), extinction (light grey) and empty (white).

The *S. avenae* population peaked on sampling occasion 14 (30 June) at just under 0.6 aphids shoot^−1^ with a subsequent gradual decline (Figure 2), and the proportion of shoots infested reached a maximum of approximately 0.25. Field scale spatial pattern gradually strengthened over the sampling period and consistent patch (high count) and gap (low count) neighbourhoods emerged in the latter stages of the growing season from sampling occasion 17 (7 July) onwards (Figures 3 and 4). Temporal stability of this field-scale spatial structure was confirmed by the SADIE local association analysis (Figure 5); from sampling occasion 10 (19 June) onwards, local counts were strongly and consistently associated with subsequent ones, indicating that spatial pattern had stabilised within the field. As the field population developed, an increasing proportion of sampling locations had stable resident populations, which exceeded 80% at the population peak (Figure 6). Earlier in the sampling sequence a substantial majority of sampling occasions were unoccupied by *S. avenae*, and considerable turnover (i.e. extinction and colonisation) was evident. Occupancy of individual shoots across the field was generally low, with stability not exceeding 10% on any sampling occasion. Colonisation and extinction events were evident, indicating a high degree of turnover at the scale of individual shoots.

**Figure 7:**
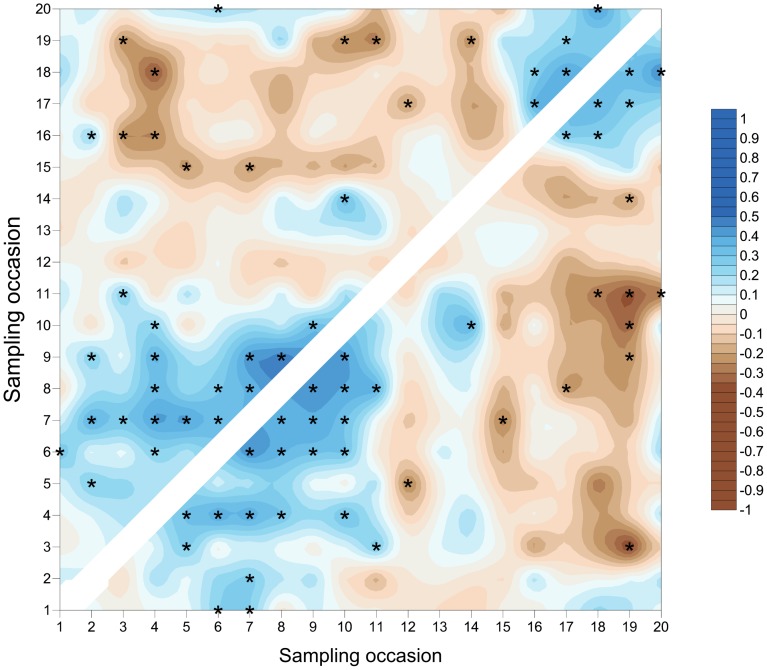
SADIE local association plot for *M. dirhodum* in Field A. Plot represents strength of local (pairwise) association between sampling occasions; blue colouring represents association and brown dissociation respectively. Significant local association at 5% is represented by asterisks. Comparisons assessed using counts (above diagonal) and proportion of shoots infested (below diagonal).

**Figure 8:**
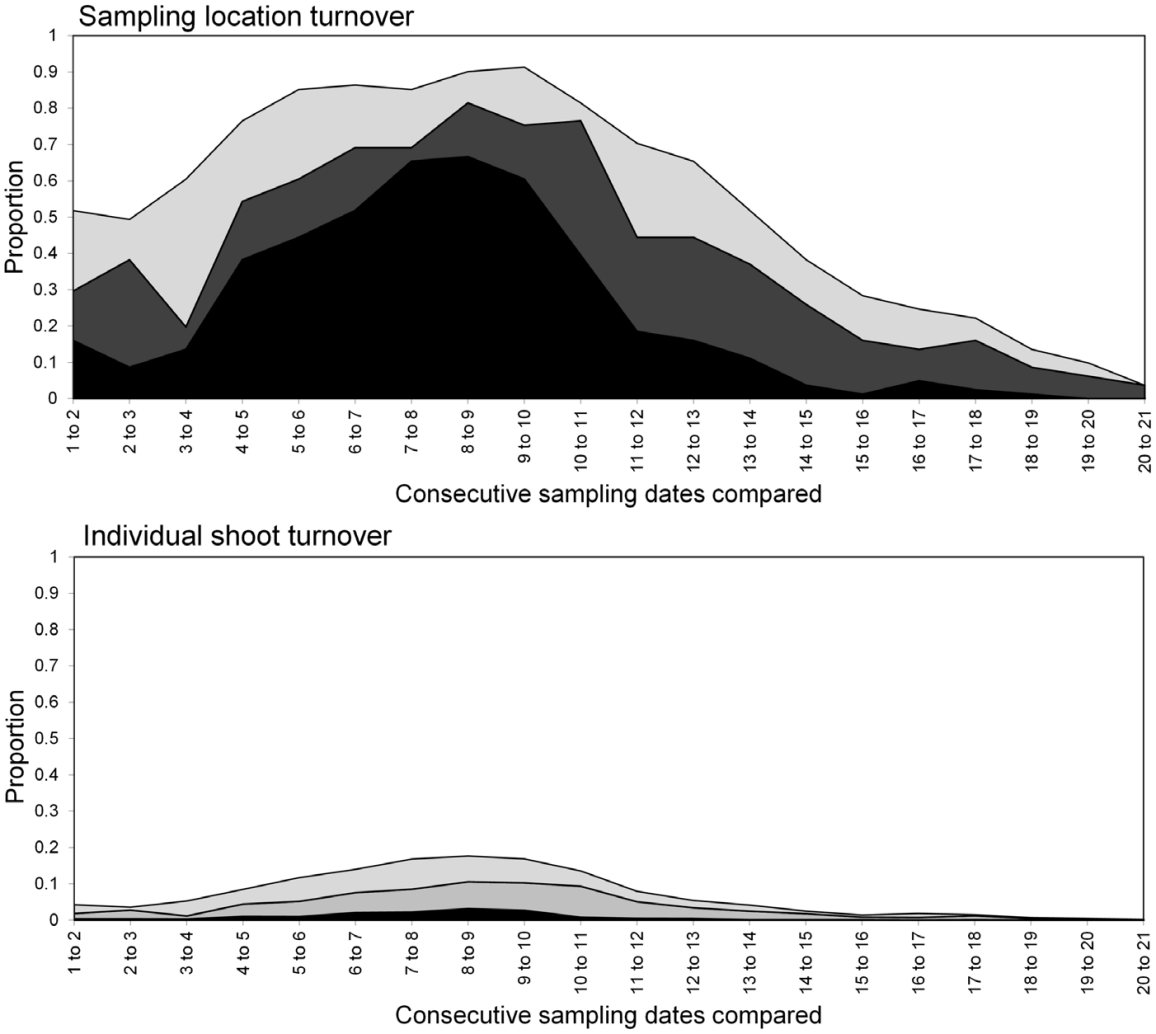
Turnover of *M. dirhodum* in Field A at sampling locations (upper) and individual shoots (lower) respectively. States represented as: stable (black), colonised (dark grey), extinction (light grey) and empty (white).

The *M. dirhodum* population established and developed earlier in the sampling period than *S. avenae*. The population also peaked earlier, on sampling occasion 9 (16 June), with a density of just over 0.3 aphids shoot^−1^, the peak proportion of shoots infested being approximately 0.1 (Figure 2). A rapid decline in population was then observed, with a very low *M. dirhodum* population evident during the latter phases of sampling. Some field-scale spatial pattern emerged between sampling occasions 8 and 10 (13 June to 19 June) coincident with peak infestation (Figures 3 and 4). Spatial pattern detected at the end of the season was anomalous, due to a very small but spatially concentrated aphid population. SADIE local association analysis indicated that some stable temporal spatial pattern within the field emerged coincident with the population peak (Figure 7), but that this was typically only evident for one or two sampling occasions. Compared with *S. avenae*, high rates of turnover (colonisation and extinction) were observed with less population stability (Figure 8). Stability did not exceed 60% at peak population, with turnover events affecting over 20% of sampling locations. Very high rates of turnover were observed at the scale of an individual shoot; only approximately 3% of the sampled shoots had stable colonies over consecutive sampling occasions when the *M. dirhodum* population peaked.

### Field B

**Figure 9:**
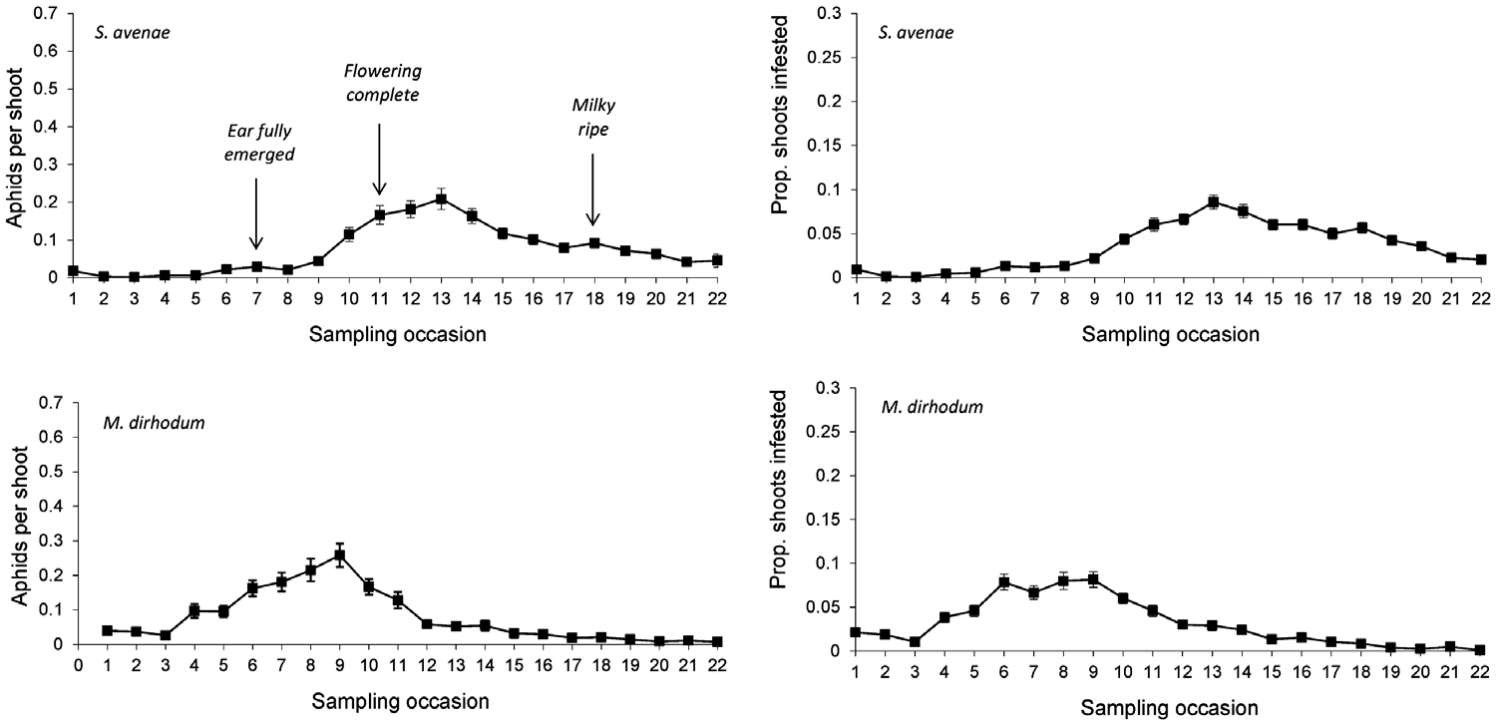
Population growth of *S. avenae* and *M. dirhodum* observed in Field B, summarised as aphids per shoot (mean ± 1 s.e.) and as proportion of shoots infested. Sampling occasions were: **1**, 19 May; **2**, 22 May; **3**, 26 May; **4,** 31 May; **5**, 2 June; **6**, 5 June; **7**, 9 June; **8**, 12 June; **9**, 16 June; **10**, 19 June; **11**, 21 June; **12**, 23 June; **13**, 26 June; **14**, 28 June; **15**, 30 June; **16**, 3 July; **17**, 5 July; **18**, 7 July; **19**, 10 July; **20**, 14 July; **21**, 17 July; **22**, 21 July.

**Figure 10:**
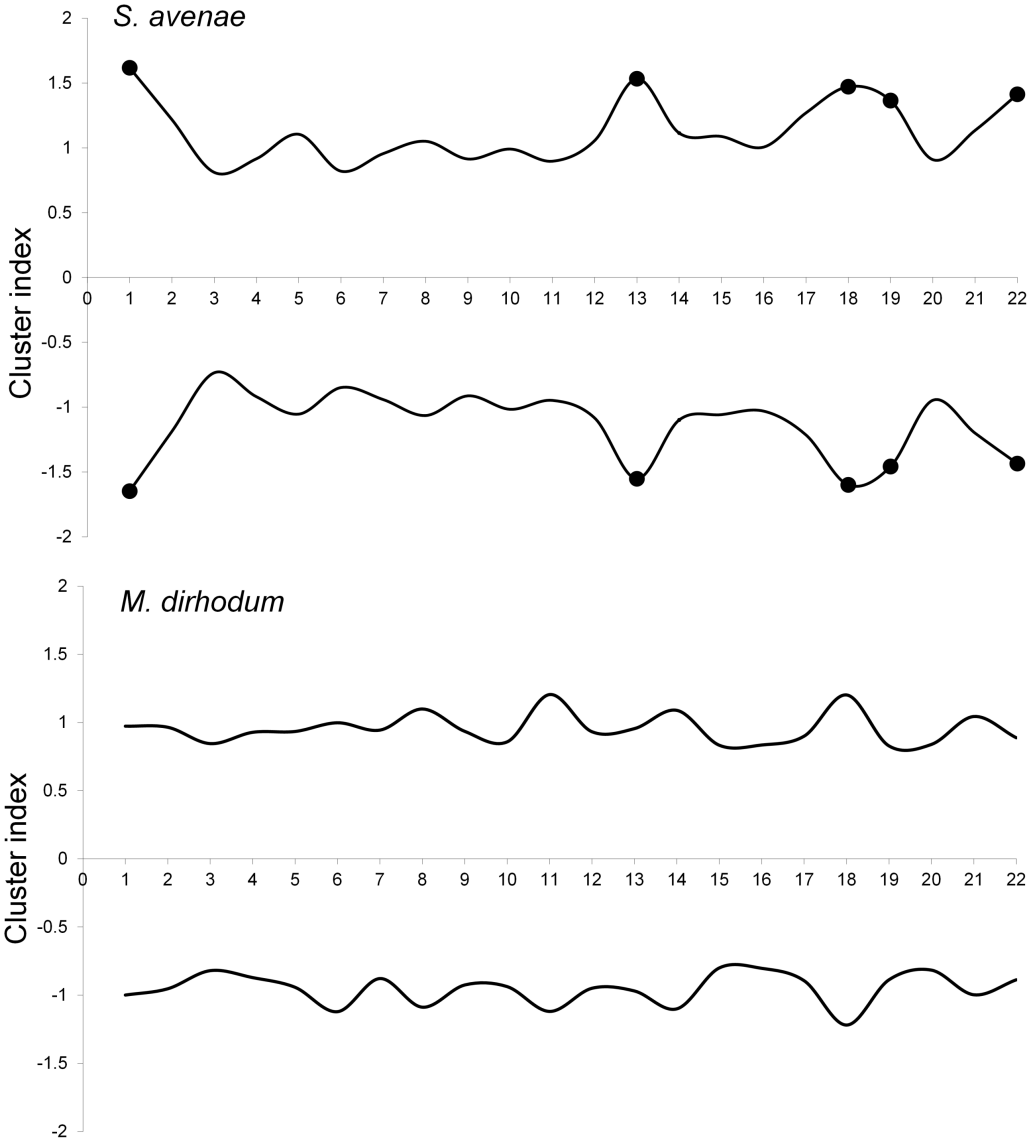
SADIE analysis representing patch (positive) and gap (negative) cluster analyses for *S. avenae* and *M. dirhodum* recorded in Field B. Filled circles represent sampling occasions when cluster indices were significant at 5% and represented measurable spatial pattern.

**Figure 11:**
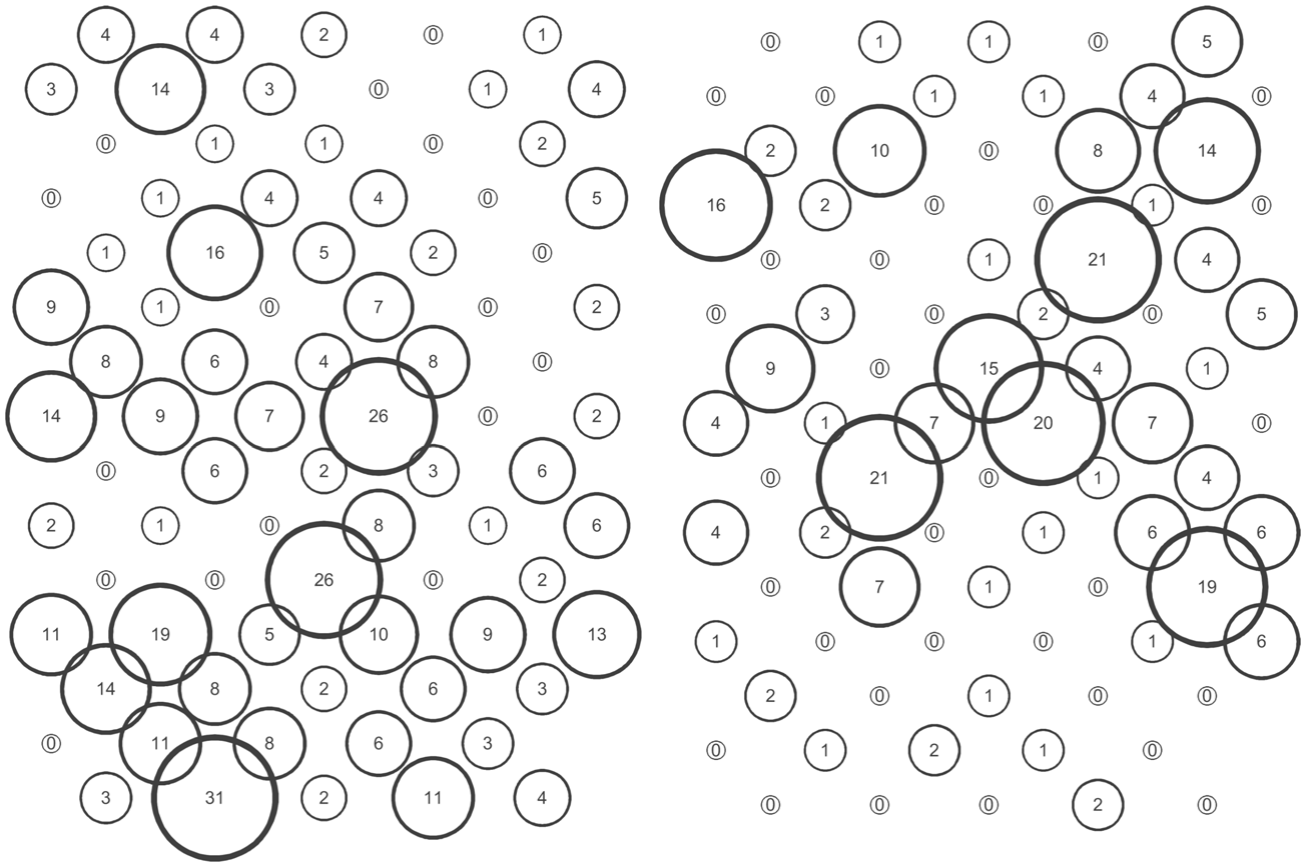
Indicative distributions in Field B when field-scale spatial pattern was most strongly expressed for *S. avenae* (sampling occasion 13, left) and *M. dirhodum* (sampling occasion 11, right).

**Figure 12:**
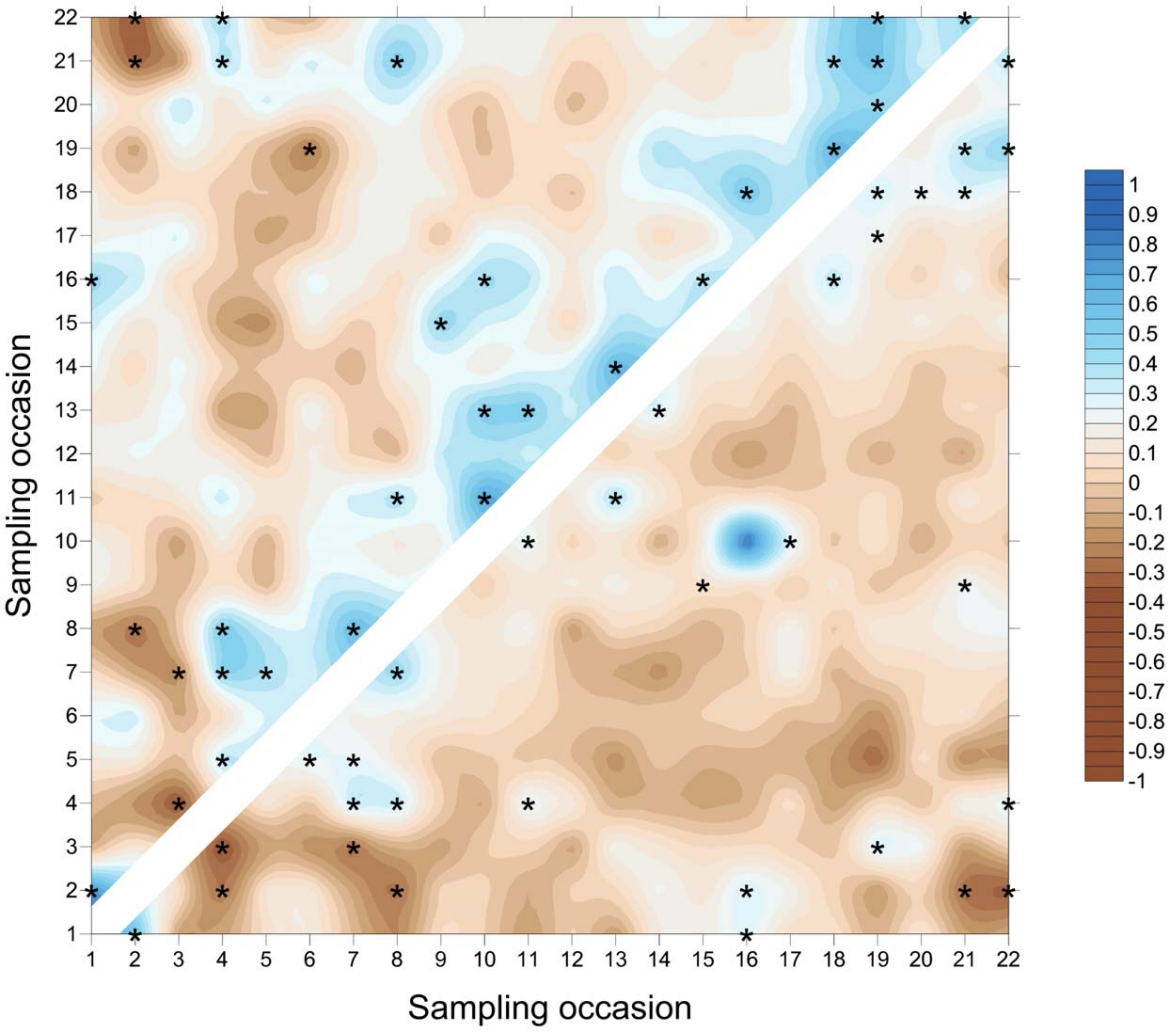
SADIE local association plot for *S. avenae* in Field B. Plot represents strength of local (pairwise) association between sampling occasions; blue colouring represents association and brown dissociation respectively. Significant local association at 5% is represented by asterisks. Comparisons assessed using counts (above diagonal) and proportion of shoots infested (below diagonal).

**Figure 13:**
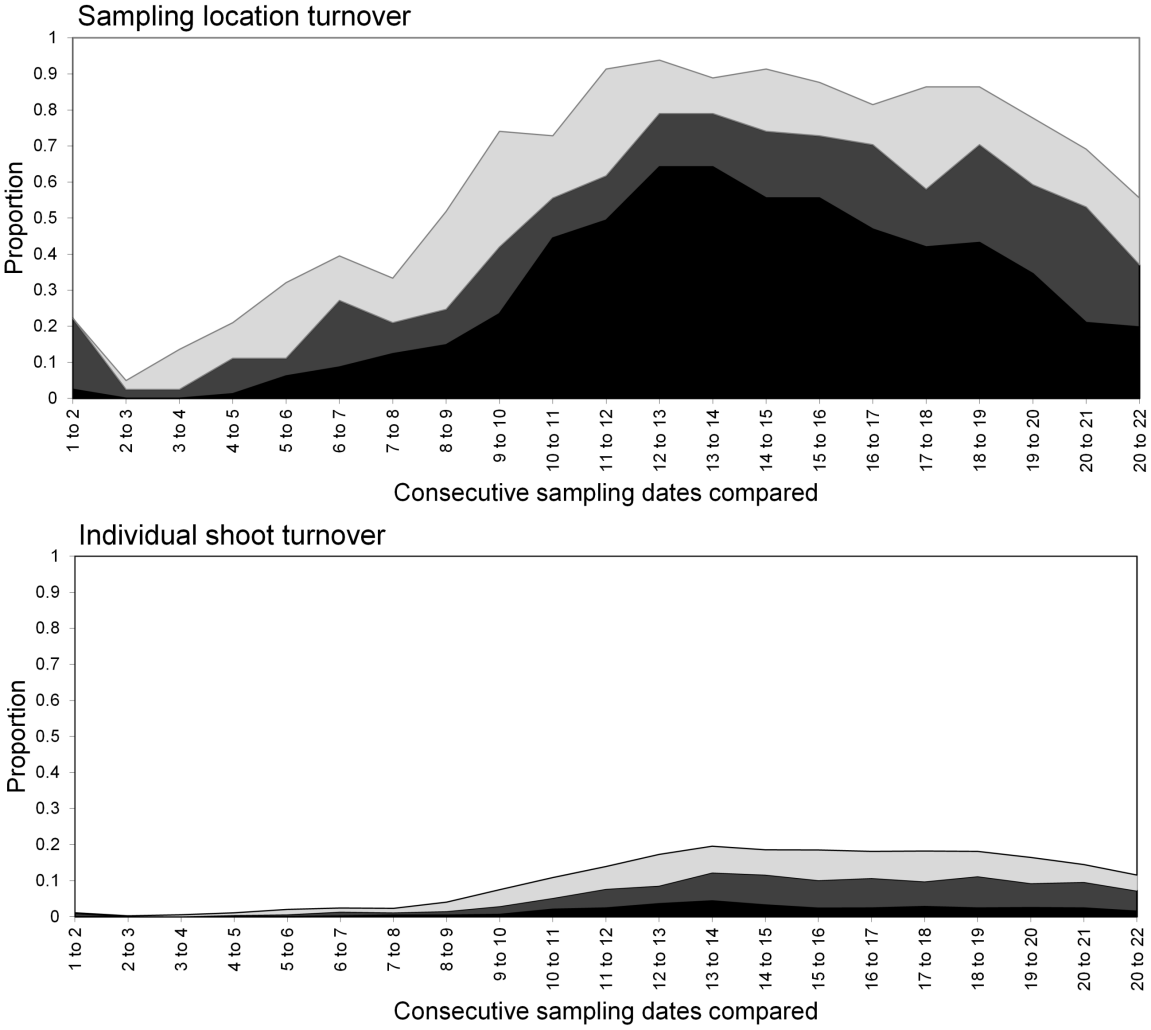
Turnover of *S. avenae* in Field B at sampling locations (upper) and individual shoots (lower) respectively. States represented as: stable (black), colonised (dark grey), extinction (light grey) and empty (white).

The *S avenae* population was substantially lower than that observed in Field A (Figure 9), peaking at 0.2 aphids shoot^−1^ but reaching its maximum at a similar date (sampling occasion 13, 26 June). The proportion of shoots infested reached a maximum of approximately 0.1 at peak infestation. A subsequent slow decline in the population was then observed. There was limited evidence of consistent field-scale spatial pattern during the sampling period, although some measurable but sporadic pattern was evident from sampling occasion 13 (26 June) onwards (Figure 10 and 11). Spatial pattern detected at the start of the sampling period was due to a single spatially concentrated aphid population. SADIE local association analysis (Figure 12) provided evidence of some short-term local association; spatial pattern was generally only associated with that observed on the prior sampling occasion (i.e. significant values closely followed the diagonal). At the population peak, about 65% of sampling locations exhibited stability, with turnover (extinction or colonisation) evident at about 25% of locations (Figure 13). Similar to Field A, very low levels of stability were observed at the scale of the individual shoot (Figure 13); the proportion of individual shoots with stable colonies did not exceed 5% throughout the sampling period.

**Figure 14:**
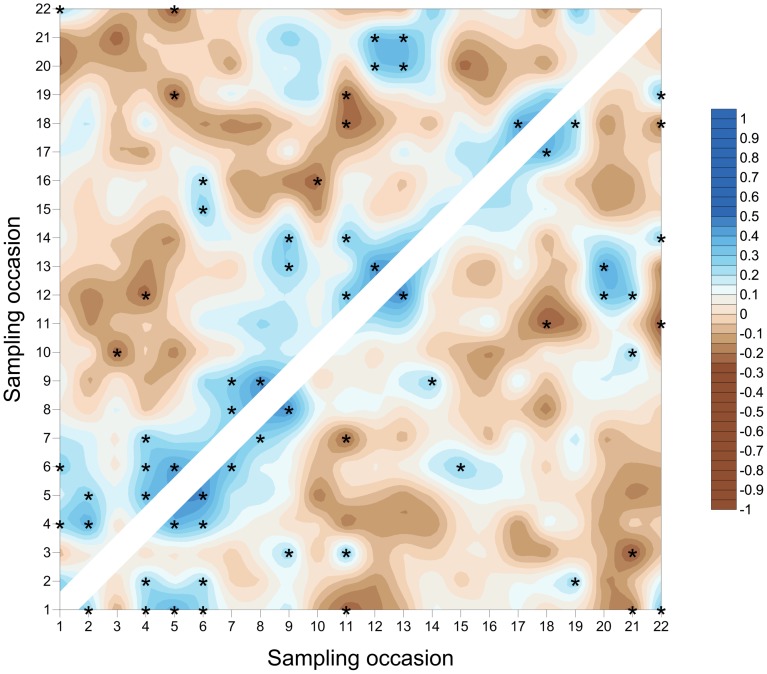
SADIE local association plot for *M. dirhodum* in Field B. Plot represents strength of local (pairwise) association between sampling occasions; blue colouring represents association and brown dissociation respectively. Significant local association at 5% is represented by asterisks. Comparisons assessed using counts (above diagonal) and proportion of shoots infested (below diagonal).

**Figure 15:**
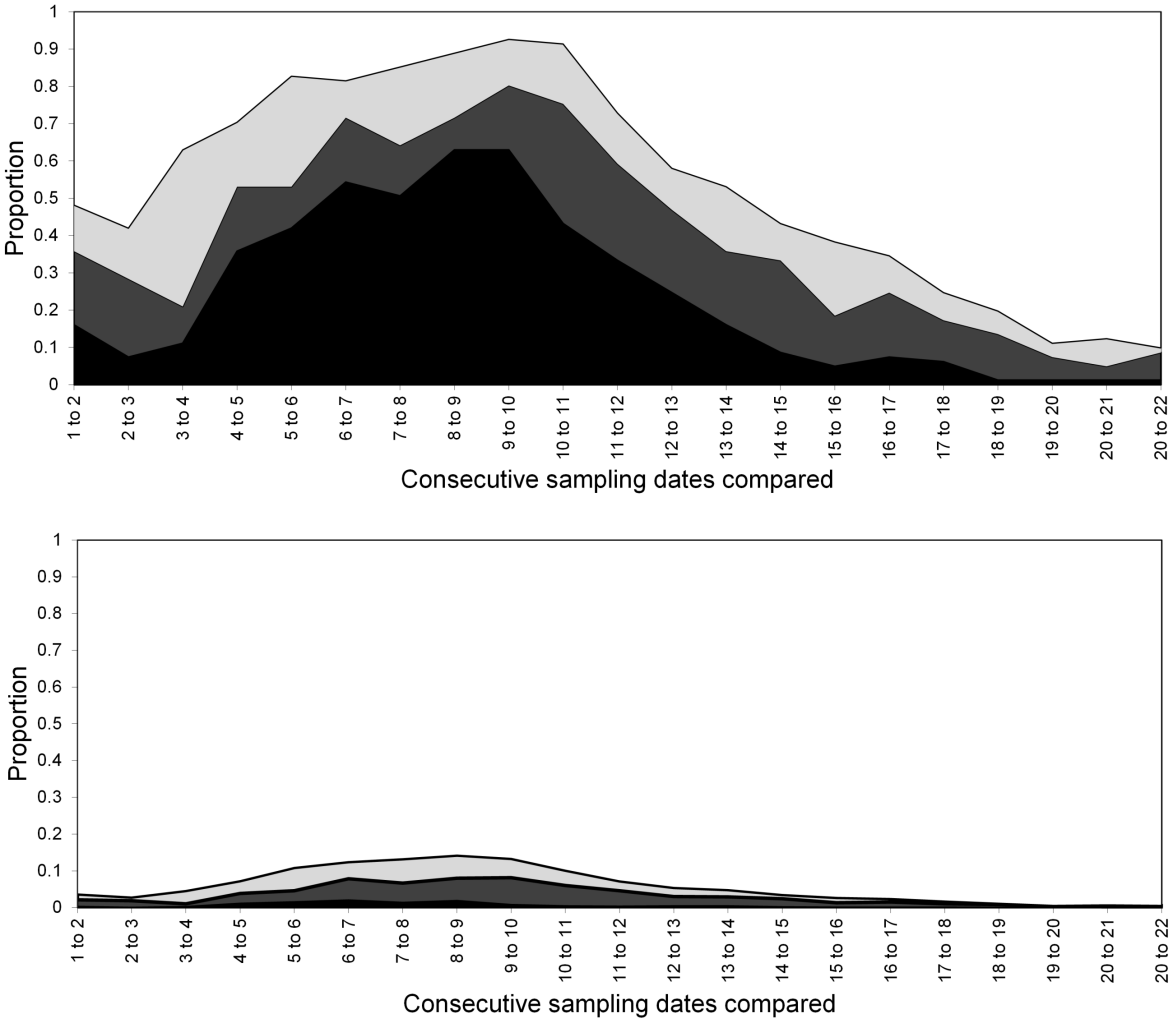
Turnover of *M. dirhodum* in Field B at sampling locations (upper) and individual shoots (lower) respectively. States represented as: stable (black), colonised (dark grey), extinction (light grey) and empty (white).

The population development of *M. dirhodum* was generally similar to that observed in Field A; peaking at approximately 0.25 aphids shoot^−1^ with infestation levels of 0.8 (Figure 9). A rapid population decline was then evident, with very low densities towards the end of the season. Although no measurable field-scale spatial pattern emerged (Figures 10 and 11), SADIE local association analysis indicated that there was some short-term population persistence evident between consecutive sampling occasions (Figure 14). A comparatively low proportion of sampling locations exhibited stability (60% at peak population) with turnover (colonisation and extinction) being typically 25% (Figure 15). Very high rates of turnover were again observed at the scale of an individual shoot, with only up to about 2.5% of shoots having a stable colony over two consecutive sampling occasions.

## Discussion

This study provided insights into the spatial and temporal development of cereal aphid populations. Using marked shoots, our intensive field study enabled us to investigate spatial processes at both the field scale and that of the individual shoot. At the scale of the individual shoot colonies of aphids were extremely ephemeral; this implies that at the small (<1 m) scale the resident aphid population is being constantly redistributed between shoots through dispersal [32]. The mechanism for this dispersal is likely to be through aphid fall-off [24], [25], [26] mediated by mechanical disturbance or attack by natural enemies [33], [34]. Dispersal by flight is limited during the cereal growing season as the adult population is dominated by flightless apterous individuals. Sopp et al. [25] showed that up to 90% of falling aphids could return to the crop within a short timescale (<10 minutes), yet there must be an energetic and physiological cost associated with this movement which possibly causes a net reduction in the rate of population increase.

This dispersal must also expose aphids to ground-active predators [35]–[37], although this may be offset by protection (through avoidance) from attack by plant-active predators. As shoot occupancy was low, individuals moving between shoots are most likely to colonise one that is unoccupied, possibly providing a partial refuge from predation or parasitism [38]. The costs and benefits of aphid movement in relation to escape and exposure to predators as well as energetic costs warrants further study. Additionally, aphids inhabiting different parts of the plant may have different vulnerabilities to physical disturbance (i.e. *S. avenae* typically reside within the ear whilst *M. dirhodum* inhabits lower leaves). Aphids on the lower leaves may also be more exposed to attack by largely epigeal Carabidae and Araneae, alongside syrphid larvae that migrate from the base of the plant to upper parts during darkness.

At the field (i.e. sampling location) scale, aphid populations were much more stable, and generally increasingly so, as the sampling period progressed. Stability was probably due to individual aphids (with limited dispersal ability) re-colonising shoots close to the sampling location. Predators and parasites within the vicinity would need searching strategies to exploit this localised yet ephemeral population if aphid consumption was maximised for the purposes of pest control.

At the field-scale, detectable spatial pattern was evident most strongly in Field A and to some extent in Field B. In general, spatial pattern was more evident later in the sampling sequence when populations had developed and it was likely that our ability to detect spatial pattern was to some extent dependent on aphid density. It is known that spatial pattern can mediate aggregative numerical response for ground-active predators [13], [39], [40] whilst spiders [12], [36] also respond by building webs in locations of higher prey abundance. The delivery of the ecosystem service of biological control is mediated by the searching strategies of such natural enemies, yet generally little is known about how they may respond within a field over a range of spatial scales [41].

Optimisation of conservation biological control strategies using a community of natural enemies may be possible if more was known regarding such responses [42]–[44]. For example, it is likely that predation and parasitism is most effective in terms of pest suppression early in the growing season (before pest populations are fully established), at a time when limited field-scale spatial pattern is evident [45]. In such a case, increasing the availability of non-crop habitats [46], [47] and adopting husbandry practices that maximise the densities of natural enemies [48] early in the season may be effective. Strategies could also focus on increasing the abundance of highly mobile predators with efficient searching strategies.

Within-field spatial pattern is well documented and models have been developed [49]–[50], but, the drivers for this are not fully understood and are undoubtedly complex. We observed differences between the two fields particularly with respect to the development of *S. avenae* populations (although the fields were within 1 km of each other and received identical husbandry practices). Drivers for such differences include initial conditions established during the immigration of winged aphids, the establishment of populations from field-resident aphids, microclimatic and topographical differences, differences in soil fertility influencing crop growth, and differences in the impact of natural enemies [7], [32], [51].

Spatial pattern of insects is difficult to describe and to do so requires considerable sampling effort. This intensive field scale study provided some insights and further information regarding the distribution of aphids in a relatively simple monocultural crop habitat. Fievet et al [7] discuss the problem of selecting an appropriate sampling scale and identifies the need to study processes at the local spatial scale before scaling up. This is particularly important in relation to ecosystem service delivery because it is at the local scale that ecosystems actually function.

## Supporting Information

Table S1 **Aphid count data for Field A.**
(XLS)

Table S2 **Aphid count data for Field B.**
(XLS)
